# Bavachin suppresses proliferation of laryngopharyngeal cancer by regulating the STAT3 and MAPK signaling pathways

**DOI:** 10.7150/jca.92956

**Published:** 2025-03-31

**Authors:** Xiaonan Yang, Zhimin Ding, Hongting Hua, Ruijia Gan, Dongdong Meng, Yan Zang, Han Xiao, Dong Wang, Wanjin Jiang, Dongyu Si, Xiang Wei, Mei Zhang, Huabing Zhang, Chaobing Gao

**Affiliations:** 1 Department of Otorhinolaryngology Head and Neck Surgery, The First Affiliated Hospital of Anhui Medical University, Hefei, 230022, China.; 2 Department of Biochemistry & Molecular Biology, Metabolic Disease Research Center, School of Basic Medicine, Anhui Medical University, Hefei, 230022, China.; 3 Health Management Center, First Affiliated Hospital of the University of Science and Technology of China (Anhui Provincial Hospital), Hefei, 230022, China.; 4 Department of Otorhinolaryngology Head and Neck Surgery, The People's Hospital of Xuancheng City, Xuancheng, 242000, China.; 5 Department of Otorhinolaryngology Head and Neck Surgery, Fuyang Women and Children's Hospital, Fuyang, 236000, China.; 6 Department of Otorhinolaryngology Head and Neck Surgery, The Fourth Affiliated Hospital of Anhui Medical University, Hefei, 230022, China.; 7 Department of Otorhinolaryngology Head and Neck Surgery, The First Affiliated Hospital of Wannan Medical College, Wuhu, 241000, China.

**Keywords:** Laryngopharyngeal cancer, Bavachin, Cell cycle, Apoptosis, MAPK, STAT3

## Abstract

**Purpose:** The present study aimed to explore the underlying antitumor effects of bavachin on laryngopharyngeal cancer *in-vitro* and *in-vivo*.

**Methods:** Tu212 and FaDu cells were cultured in the incubator. Cells were treated with 0.1% DMSO (control group) and different concentrations of bavachin (experimental groups) for exploring the results of proliferation and apoptosis. We revealed the underlying mechanism of bavachin on laryngopharyngeal cancer through western blotting, qRT-PCR assay and immunofluorescence staining.

**Results:** Bavachin could suppress the proliferation and migration of laryngopharyngeal cancer cells *in-vitro* and *in-vivo*. Mechanistically, the results suggested that bavachin could downregulate the phosphorylation level of the signal transducer and activator of the transcription 3 (STAT3) and upregulate those of the mitogen-activated protein kinase (MAPK). Furthermore, bavachin also increased the expression level of Bax and suppressed those of Bcl-2, CDK4/6, and CyclinD1 in the laryngopharyngeal cancer cells. Additionally, the study also identified that bavachin promoted ferroptosis by decreasing the expression level of glutathione peroxidase 4 (GPX4) and increasing those of intracellular reactive oxygen species (ROS) and glutathione (GSH).

**Conclusion:** Taken together, these results demonstrated that bavachin could suppress the growth and migration of laryngopharyngeal cancer cells and induce apoptosis and cell cycle arrest of the laryngopharyngeal cancer cells by regulating the MAPK/STAT3 signaling pathway. This study demonstrated that bavachin exhibited a clinical therapeutic potential for laryngopharyngeal cancer.

## Introduction

The incidence rate of head and neck cancer has recently been increasing, especially that of head and neck squamous cell carcinoma (HNSCC), which is the sixth most common type of cancer worldwide 1. Among the HNSCC, laryngopharyngeal carcinoma is the second most common type of cancer. It occurs in the laryngopharyngeal mucosal epithelium and accounts for approximately 90% of laryngopharyngeal carcinoma, which caused the death of 99,840 people in 2020 worldwide 2-4. The symptoms of laryngopharyngeal cancer at the early stage are not obvious; therefore, approximately 60% of the patients are diagnosed in the late stages (III or IV), in which, the patients experience severe impairment of vocal, respiratory, and swallowing function 5. Although the therapeutic strategies for laryngopharyngeal cancer have considerably progressed over the years, surgical resection remains the core treatment strategy 6. However, surgery cannot guarantee the patient's survival and quality of life and its five-year survival rate has decreased from 66% to 63% due to unexplained pathogenesis and development 7. Therefore, chemotherapy has been focused on due to its protective effects on laryngopharyngeal function 8.

**Figure 1:**
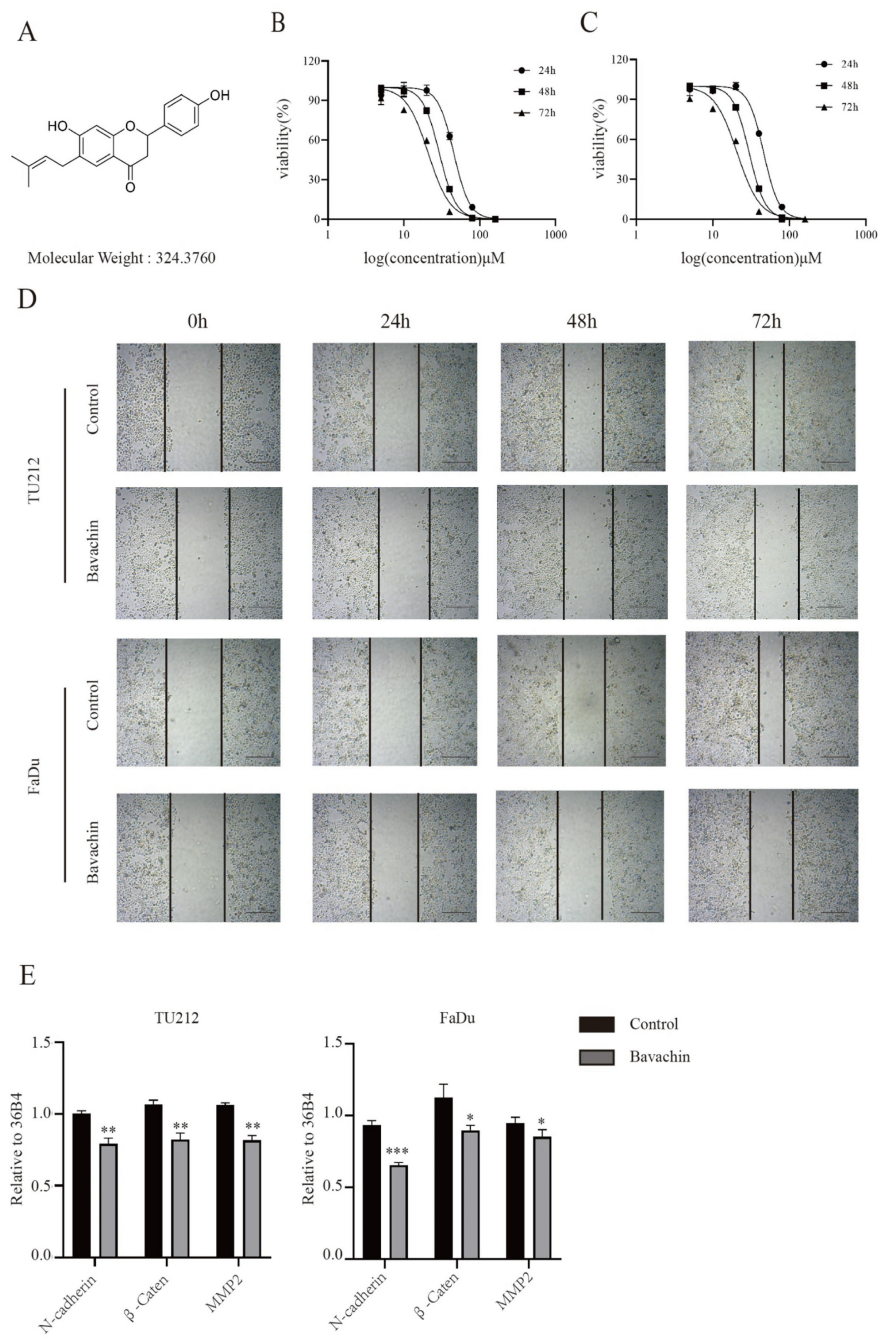
Bavachin suppressed the viability of laryngopharyngeal cancer cells and inhibited the migration of laryngopharyngeal cancer cells. (A) Chemical structure of bavachin. (B-C) MTT assays showed that bavachin (5, 10, 20, 40, 80, 160 μM/L for 24, 48 and 72h, respectively) decreased the viability of laryngopharyngeal cancer cells (n = 3). (D) Wound healing assays showed that bavachin (20 μM) significantly obstructed the migration ability of Tu212 and FaDu cells (n = 3). (E) qPCR analysis of the genes involved in migration of Tu212 and FaDu cells after bavachin treatment (n = 3). *P <0.05, **P <0.01, ***P <0.001.

Studies on and uses of herbal medicines have increased worldwide. Bavachin is a flavonoid extracted from the fruit of *Psoralea corylifolia.* Its chemical structure is shown in Figure 1A, its molecular formula is C_20_H_20_O_4_, and its molecular weight is 324.37. It has various functional activities such as anti-inflammatory 9, antiviral 10, anti-tumor 11, promoting osteogenesis and immune regulation^12^,^13^, and also plays an important role in skin diseases 14.

Bavachin can improve asthma inflammation by inhibiting the expression of phospho-STAT6 and GATA-3 15. It could also inhibit the growth of various tumor cells by inducing apoptosis via interfering with mitochondria to regulate the endoplasmic reticulum stress pathway in HepG2 cells 16. Moreover, bavachin could cause ferroptosis in the osteosarcoma cells via STAT3/P53/SLC7A11 axis 17. However, the therapeutic effects of bavachin on human laryngopharyngeal cancer cells have not been studied.

The current study showed that bavachin could mediate the cellular proliferation and migration, cell cycle arrest, apoptosis, and ROS activity of Tu212 and FaDu cells by regulating the MAPK/STAT3 pathway *in-vitro*. Furthermore, its effects on tumor formation in nude mice were also explored by injecting the tumor cells *in-vivo*. Bavachin might have the potential for the clinical treatment of laryngopharyngeal tumors.

## Materials and methods

### Reagents

Human laryngopharyngeal squamous cell carcinoma (Tu212) cells were obtained from BeNa Culture Collection (Beijing, China). Human pharyngeal squamous cell carcinoma (FaDu) cell lines were purchased from World Cell Factory (Shanghai, China). Bavachin (CS-6113, purity >99%) was obtained from MedChemExpress (MCE, Shanghai, China). The following primary antibodies were used in this study: β-actin (Cat. No. 4970), JNK (Cat. No. 9252), p-JNK (Cat. No. 4668), Stat3 (Cat. No. 4904), p-Stat3 (Cat. No. 9131), E-cadherin (Cat. No. 3195), MAPK (Cat. No. 9212), and p-MAPK (Cat. No. 4631) were purchased from Cell Signaling Technology (MA USA); CDK6 (Cat. No. 66278-1-1g), CDK4 (Cat. No. 66950-1-1g), Cyclin D1 (Cat. No. 60186-1-1g), MFN1 (Cat. No. 13798-1-AP), and MFN2 (Cat. No. 12186-1-AP) were purchased from Proteintech (Wuhan Sanying, China); Ki67 (Cat. No. A2094) and FITC Goat Anti-Rabbit lgG (Cat. No. AS011) were purchased from ABclonal (Wuhan, China); and Cleaved-Caspase 3 (Cat. No. AF7022) and GPX4 (Cat. No. DF6701) were purchased from Affinity Biosciences (Giangsu, China).

### Cell culture

The Tu212 and FaDu cell cultures were maintained in RPMI Medium 1640 basic (Gibco, China), containing 10% fetal bovine serum (FBS, Lonsera, Australia), penicillin (100 U/ml), and streptomycin (100 mg/ml, Beyotime, China). Both the cell cultures were maintained at 37ºC in a humidified atmosphere with a 5% CO_2_ concentration.

### Cell proliferation assay

The cells were treated with 0.1% DMSO (control group) and different concentrations of bavachin (5, 10, 20, 40, 80, and 160 μmol/L) (experimental groups) for 24, 48 and 72 h. MTT solution (25 μL) was added to each well. Then, the supernatant was sucked out and 100-μL DMSO was added to each well. Finally, the optical density (OD value) of each well was measured at 490 nm using a microplate reader.

### Colony formation assay

Approximately 1500 cells were cultured into each well in a 6-well plate. After the cell adhered to the walls, the control and experimental groups were treated with 0.1% DMSO and 5, 10, and 20 μmol/L bavachin, respectively. The DMSO and bavachin were replaced every four days. On the 10^th^ day, the cells were fixed with 4% paraformaldehyde for 30 min. Then, the cells were stained with 0.1% crystalline violet staining for 30 min and observed using digital camera.

### Wound healing assay

After the monolayer cells reached the confluence state in a 6-well plate, the cells were scratched. Then, the control and experimental groups were treated with 0.1% DMSO and 20 μmol/L bavachin, respectively. The states of cell migration were observed at different times (0, 24, 48, and 72 h) using an inverted microscope.

### Apoptosis detection assay

The cells were evenly spread in the wells of a 6-well plate and incubated overnight. The cells were treated with DMSO (0.1%) and bavachin (20 μmol/L) as the control and experimental groups for 24 h, respectively. The cells were subsequently processed using Annexin V Apoptosis Detection kit (Bestbio, China, BB-4101) following the manufacturer's instructions. The cellular apoptosis results were measured using a flow cytometer. The data were analyzed using FlowJo V10 software.

### Cell cycle assay

The cell cycle was detected using propidium iodide (PI, Bestbio, China, BB-4104). When the number of cells reached 4×10^5^ per well in a 6-well plate, the cells were treated with 0.1% DMSO and 20 μmol/L bavachin as the control and experimental groups and incubated for 24 h. The cells were then collected and fixed with cold anhydrous ethanol overnight. The next day, the cells were incubated with 500-μL phosphate buffered saline (PBS), containing 20 μL Rnase A, at 37ºC for 30 min. Finally, the samples were treated with 400 μL PI and incubated in dark for 30 min to 1 h at 4ºC. The cells were transferred to the flow tube. The cell cycle results were analyzed using FlowJo V10 software.

### ROS and GSH assay

The ROS levels were analyzed using DCFH-DA (Beyotime, China, S0033S). Approximately 5×10^5^ FaDu and Tu212 cells per well were spread in a 6-well plate and cultured overnight. The cells were then treated with 0.1% DMSO and 20 μM bavachin as control and experimental groups for 24 h, respectively. The harvested cells were stained with 10 μmol/L DCFH-DA for 20 min at 37ºC, immediately washed 3 times with basal medium, and detected using a flow cytometer. The intracellular GSH levels were assessed using a GSH colorimetric assay kit (Solarbio, China, BC1175).

### Western blotting

In order to analyze the expression levels of intracellular proteins, β-actin, JNK, p-JNK, Stat3, p-Stat3, p-MAPK, CDK6, CDK4, Cyclin D1, MFN1, MFN2, GPX4, cells were lysed using RIPA buffer (Beyotime, China, P0013B). The total proteins were separated using sodium dodecyl sulfate-polyacrylamide gels (8%-12%) electrophoresis based on the molecular weight of the target protein. The separated proteins were transferred to polyvinylidene fluoride (PVDF, 0.45 μm, Millipore) membranes, which were blocked with 5% skim milk for 1 h. Next, the membranes were incubated at 4ºC with the homologous primary antibodies for 10 to 12 h, followed by four times washing with TBST buffer. After incubating with the respective secondary antibodies for 1.5 h, the target proteins were observed via ECL using a chemiluminescence apparatus.

### qRT-PCR assay

The FaDu and Tu212 cells were divided into control and experimental groups, which were treated with 0.1% DMSO and 20 μmol/L bavachin, respectively. After washing the cells twice with cold PBS, the intracellular RNA was extracted using TRIzol reagent (Ambion, USA) and reverse transcribed into cDNA using gradient thermal cycler (LongGene, China). The 20-μL PCR reaction mixture included cDNA as a template, qPCR Master Mix (TOLOBIO, China), diethyl pyrocarbonate (DEPC)-treated water (Sangon Biotech, China), and relevant primers. The PCR reaction was run on a Real-Time PCR detection system (Roche, China).

### Tumor formation assay in nude mouse

Five-week-old BALB/c male nude mice were obtained from GemPharmatech LLC (Nanjing, China) and housed in a Specific pathogen Free (SPF) environment. All the animal treatments and experiments were performed as per the guidelines of the Animal Center of Anhui Medical University and approved by the Animal Ethical Committee of Anhui Medical. The FaDu cells (4×10^5^) were resuspended in a basic medium and subcutaneously administered into the lower right flank of each mouse. The mice's weight and tumor size were measured every two. When the tumor volume reached approximately 100 mm^3^, the experimental and control group were administered 60 mg/kg of bavachin and equal volume of DMSO solvent every two days, respectively. After 3 weeks, the tumor tissues were excised from the nude mice and completely preserved for further analysis.

### Immunofluorescence staining

The tumor tissues excised from the nude mice were fixed with 4% paraformaldehyde for two days, paraffinized, and cut into 5-μm-thick sections, which were then fixed on adhesive slides. After the slides were completely dried at 65ºC, they were orderly put into xylene, ethanol, and distilled water. The antigens were restored in ethylenediamine tetraacetic acid disodium salt (EDTA) in the microwave. After cooling the slides naturally to room temperature, the restored antigens were incubated with 5% normal goat serum for 2 h at 37ºC, followed by incubation with the primary antibodies for cleaved-Caspase3 and Ki67 at 4ºC overnight. The tissue sections were allowed to reach room temperature and then incubated with the corresponding secondary antibodies at 37ºC for 1.5 h. Then, the tissue sections were stained with DAPI at room temperature for 10 min. Finally, the stained sections were observed under a fluorescence microscope.

### Histopathological analysis

For the histopathological analysis of the tumor tissues, they were immersed in 4% formalin for two days and paraffinized. The tumor tissues were cut into 5-μm-thick sections using a paraffin cutting machine and then stained with hematoxylin and eosin (H&E). Finally, the histopathology of tumor tissues was observed under a microscope.

### Statistical analyses

All the data were analyzed with the paired *t*-test and one-way analysis of variance for multiple comparisons using GraphPad Prism to estimate the statistical significance. A *P*-value of <0.05 was considered statistically significant.

## Results

### Bavachin could inhibit the proliferation of laryngopharyngeal cancer cells *in-vitro*

**Figure 2:**
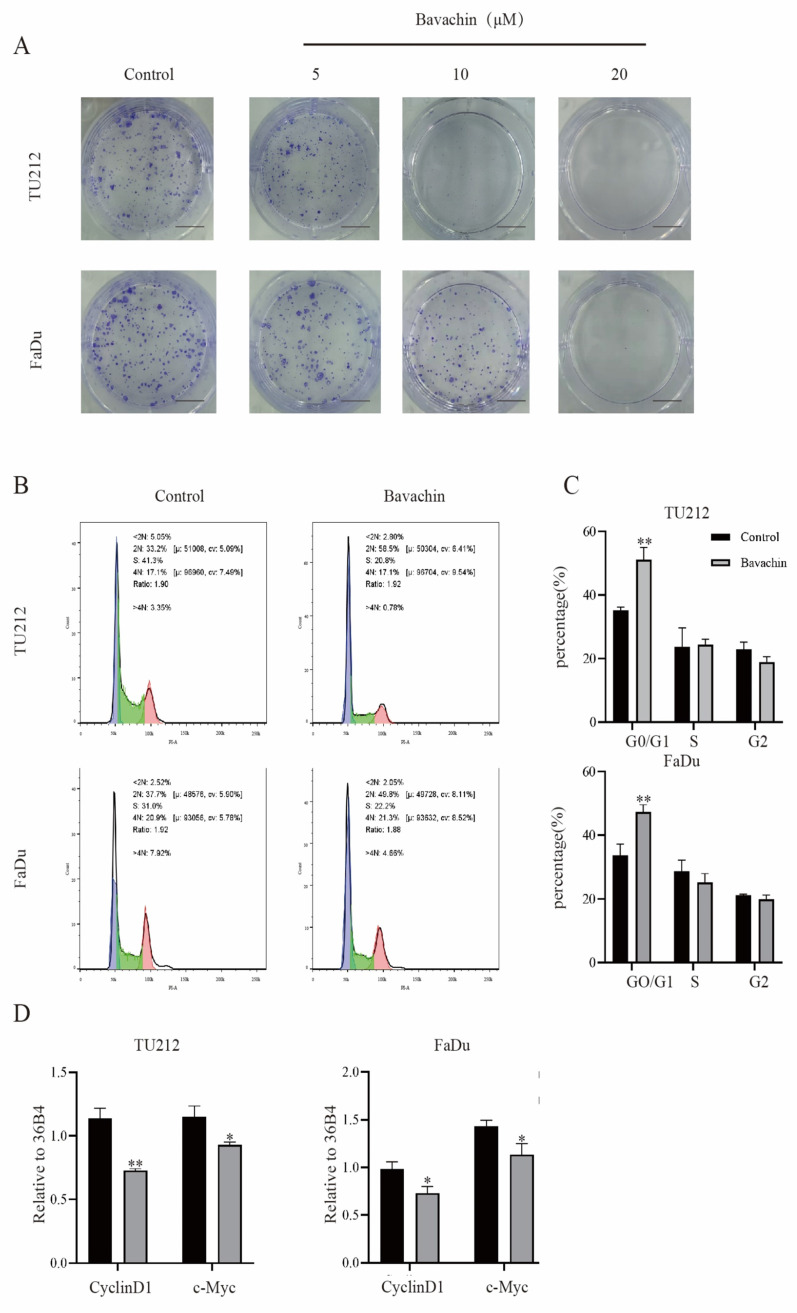
Bavachin inhibited cellular proliferation and caused cell cycle arrest of laryngopharyngeal cancer cells. (A) Effect of bavachin on colony formation (n = 3). (B-C) Laryngopharyngeal cancer cells were treated with bavachin (20 μM) for 24 h and analyzed using a flow cytometer. Bavachin induced G0/G1 cell cycle arrest as compared to the control group. (E) qPCR results of genes related to the cell cycle of Tu212 and FaDu cells after treatment with bavachin (20 μM/L) for 24 h (n = 3). *P <0.05, **P <0.01, ***P <0.001.

Previous studies indicated that bavachin could suppress the growth of a wide variety of tumors 14, 15. In order to analyze the effects of bavachin on laryngopharyngeal cancer cells, the Tu212 and FaDu cells were treated with different concentrations of bavachin. Then, the viability and proliferation ability of the cells were analyzed using MTT and colony formation assays, respectively. The MTT assay showed that the viability of Tu212 and FaDu cells gradually reduced with the increase in the concentration of bavachin. By fitting the data, the IC50 values of bavachin for Tu212 and FaDu cells were identified to be 46.09 μM and 52.26 μΜ, respectively after 24 h (Figure 1. B-C). For the colony formation assay, different bavachin concentrations (5, 10, and 20 μM/L) were selected. The colony formation ability of Tu212 and FaDu cells gradually decreased with the increase in the concentration of bavachin (Figure 2. A). The results showed that bavachin could inhibit the proliferation of laryngopharyngeal cancer cells.

### Bavachin could suppress the migration of laryngopharyngeal cancer cells *in-vitro*

A wound healing assay was performed to explore the effects of bavachin on the migration of laryngopharyngeal cancer cells. The result showed that bavachin treatment significantly inhibited the migration distance of laryngopharyngeal cancer cells as compared with the control group (Figure 1. D). Previous studies showed that the lower expression level of E-cadherin and higher expression levels of *N-cadherin*, *β-catenin*, and *MMP2* were correlated with an increase in tumor cell migration and worse prognosis 18-20. Interestingly, this study showed that the protein levels of E-cadherin increased significantly after treating the Tu212 and FaDu cells with bavachin (Figure 5. A-B). Additionally, q-RT-PCR analysis also showed that the bavachin treatment decreased the mRNA levels of *MMP2* and *N-cadherin* in the Tu212 and FaDu cells as compared to the control group (Figure 1. E). These results suggested that bavachin could suppress the migration of Tu212 and FaDu cells *in-vitro*.

### Bavachin could arrest laryngopharyngeal cancer cells in G0/G1 phase

The cell cycle assay results were analyzed using flow cytometry to determine the effects of bavachin on the progression of cell cycles. The result showed that the bavachin treatment arrested the Tu212 and FaDu cells in the G0/G1 phase as compared to the control group (Figure 2. B). The 20-μM/L bavachin treatment for 24 h significantly increased the proportion of Tu212 and FaDu cells in the G0/G1 stage as compared to the control group, and the proportion of cells in the S and G2/G0 stage was reduced (Figure 2. C). Among the stages of the cell cycle, G0/G1 phase is tightly regulated by cyclin D1, CDK4/6, and other related proteins 21. In this study, Western blot analysis also showed that the bavachin treatment lowered the expression levels of cyclin D1 and CDK4/6 in both the Tu212 and FaDu cells as compared to those of the control group (Figure 5. C-D). Moreover, the qRT-PCR also demonstrated that bavachin treatment decreased the expression levels of *Cyclin D1* and *c-Myc* (Figure 2. D). These results suggested that bavachin could arrest the Tu212 and FaDu cells in G0/G1 phase.

### Bavachin could promote the apoptosis of laryngopharyngeal cancer cells *in-vitro*

**Figure 3:**
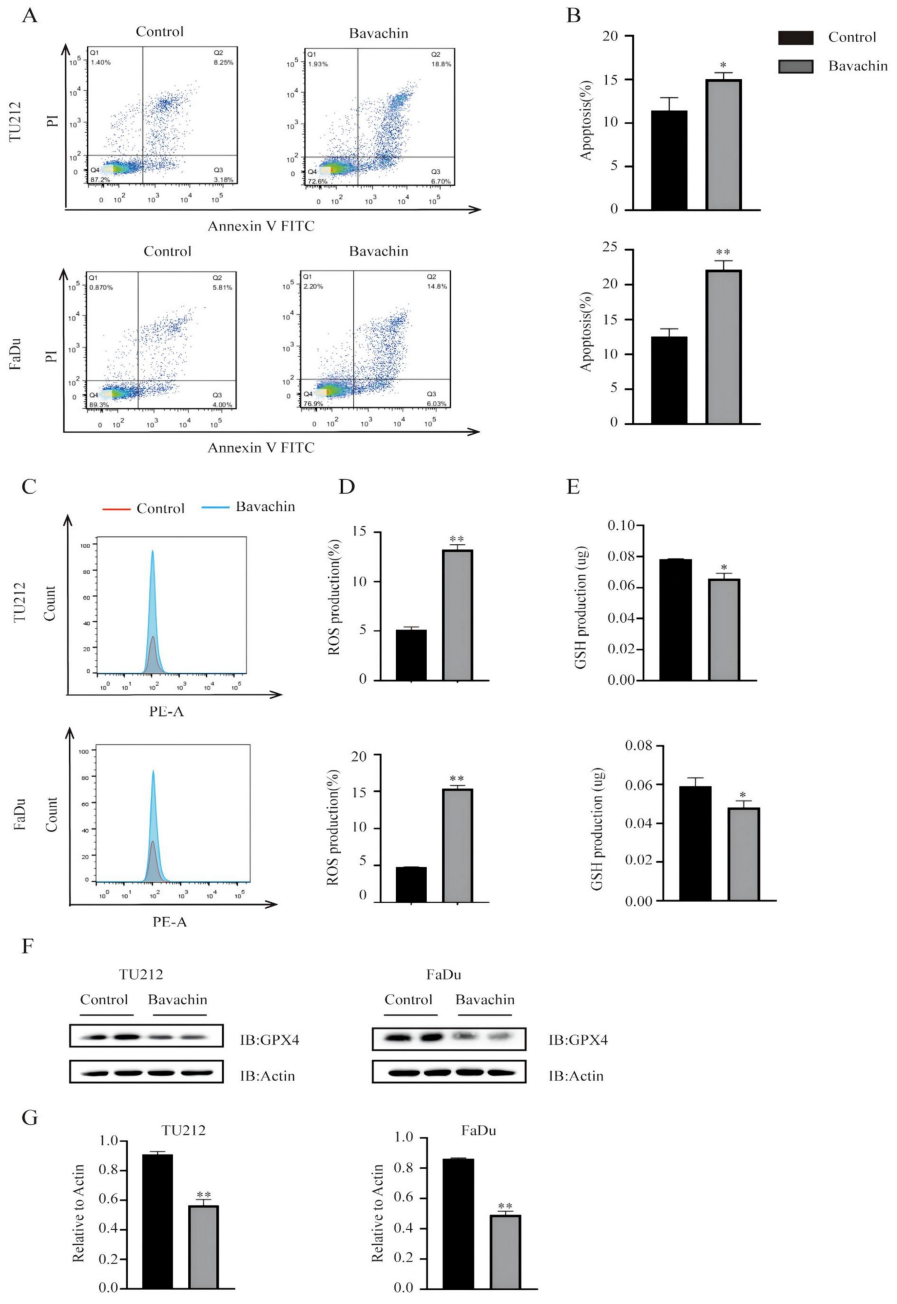
Bavachin induced the apoptosis of laryngopharyngeal cancer cells. (A-B) Bavachin (20 μM/L) significantly induced the apoptosis of laryngopharyngeal cancer cells (25.5% in the bavachin treatment group *vs.* 11.43% in the control group in the Tu212 cells and 20.83% in the bavachin treatment group *vs.* 9.81% in the control group in the FaDu cells, respectively) after 24 h (n = 3). (C-D) Bavachin (20 μM/L) promoted the accumulation of ROS in Tu212 and FaDu cells after 24 h (n = 3). (E) Bavachin (20 μM/L) treatment after 24 h decreased the intracellular level of GSH in the Tu212 and FaDu cells (n = 3). (F) Bavachin (20 μM/L) treatment remarkably suppressed the expression levels of GPX4 in the Tu212 and FaDu cells after 24 h (n = 3). (G) Statistical results of the protein expression indicated in F. *P <0.05, **P <0.01.

The apoptosis detection assay results were analyzed using flow cytometry to explore the effects of bavachin on the apoptosis of Tu212 and FaDu cells. The results showed that bavachin significantly increased the early and late apoptotic rate of Tu212 and FaDu cells. In the Tu212 cells, the experimental (20 μM/L bavachin) and control groups showed cellular apoptosis of 25.5% and 11.43%, respectively, while those in the FaDu cells were 20.83% and 9.81%, respectively (Figure 3. A-B). The mechanism of the bavachin-induced apoptosis was further elucidated by analyzing the alterations in apoptosis-related proteins using Western blotting. These results showed that the expression level of BAX was higher in the experimental group as compared to the control group (Figure 5. A-B). Meanwhile, the expression level of Bcl-2 decreased in the experimental group in both the Tu212 and FaDu cells (Figure 5. A-B). These results demonstrated that bavachin could promote the apoptosis of the laryngopharyngeal cancer cells.

### Bavachin could promote ferroptosis by accumulating ROS and consuming GSH regulated by MAPK and STAT3

**Figure 4:**
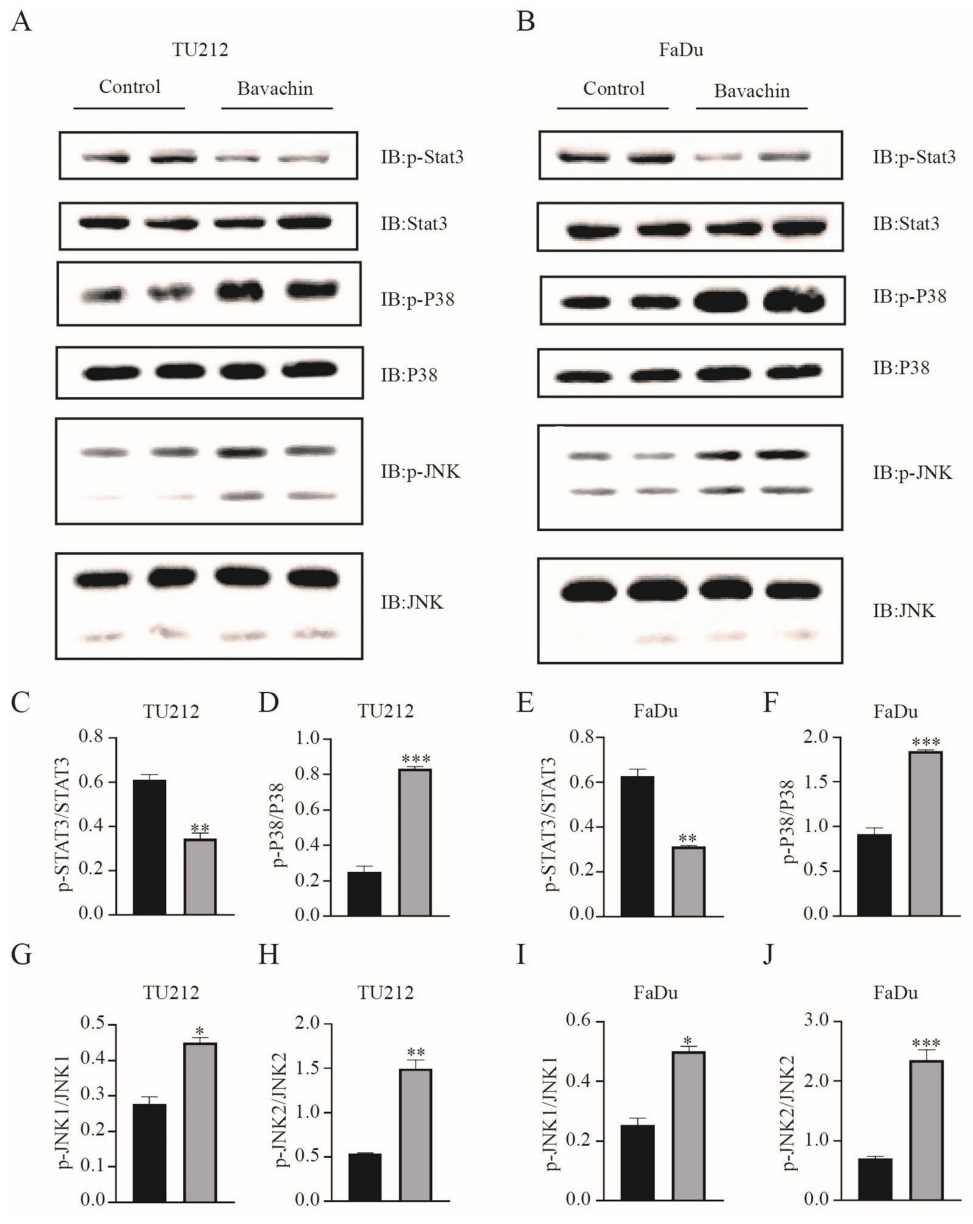
Bavachin (20 μM/L) treatment remarkably suppressed the expression levels of p-STAT3 and promoted the phosphorylation levels of P38 and JNK in the Tu212 and FaDu cells after 24 h (n = 3). (C-J) Statistical results of the protein expression indicated in A and B. *P <0.05, **P <0.01, ***P <0.001.

**Figure 5:**
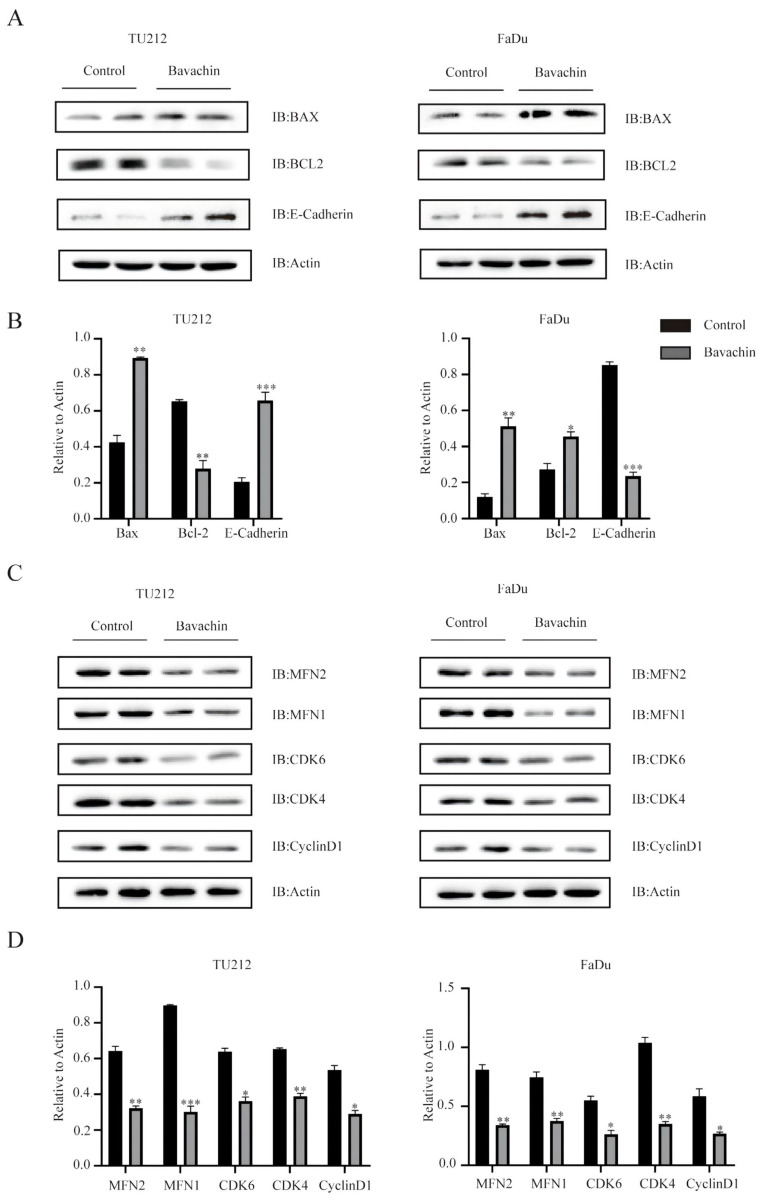
(A) Bavachin (20 μM/L) downregulated the protein expression level of Bcl-2 and upregulated those of Bax and E-cadherin in the Tu212 and FaDu cells. (B) Statistical results of the protein expression indicated in A. (C) Western blot results of protein related to the cell cycle of Tu212 and FaDu cells after treatment with bavachin (20 μM/L) for 24 h. (D) Statistical results of the protein expression indicated in C. *P <0.05, **P <0.01, ***P <0.001.

A previous study demonstrated that bavachin could induce ferroptosis in osteosarcoma cells by regulating the STAT3/P53/SLC7A11 signaling pathway 15. Therefore, this study explored the effects of bavachin on inducing ferroptosis in the Tu212 and FaDu cells. The results showed that the bavachin treatment increased the level of intracellular ROS (13.6% and 15.7%) as compared to the control group (4.88% and 4.81%) in the Tu212 and FaDu cells, respectively (Figure 3. C-D). The bavachin treatment also increased the consumption of intracellular GSH as compared to the control group (Figure 3. E). The expression levels of the key pathway components of Tu212 and FaDu cells were analyzed using Western blotting. The results showed that the bavachin treatment suppressed the phosphorylation level of STAT3 and promoted that of P38 and JNK (Figure 4. A-B). Meanwhile, the bavachin treatment downregulated the expression of GPX4 in the Tu212 and FaDu cells (Figure 3. F). These results demonstrated that bavachin could promote ferroptosis by increasing the level of intracellular ROS and inhibiting the expression levels of GPX4 and GSH.

### Bavachin could inhibit the growth of laryngopharyngeal cancer cells *in-vivo*

**Figure 6:**
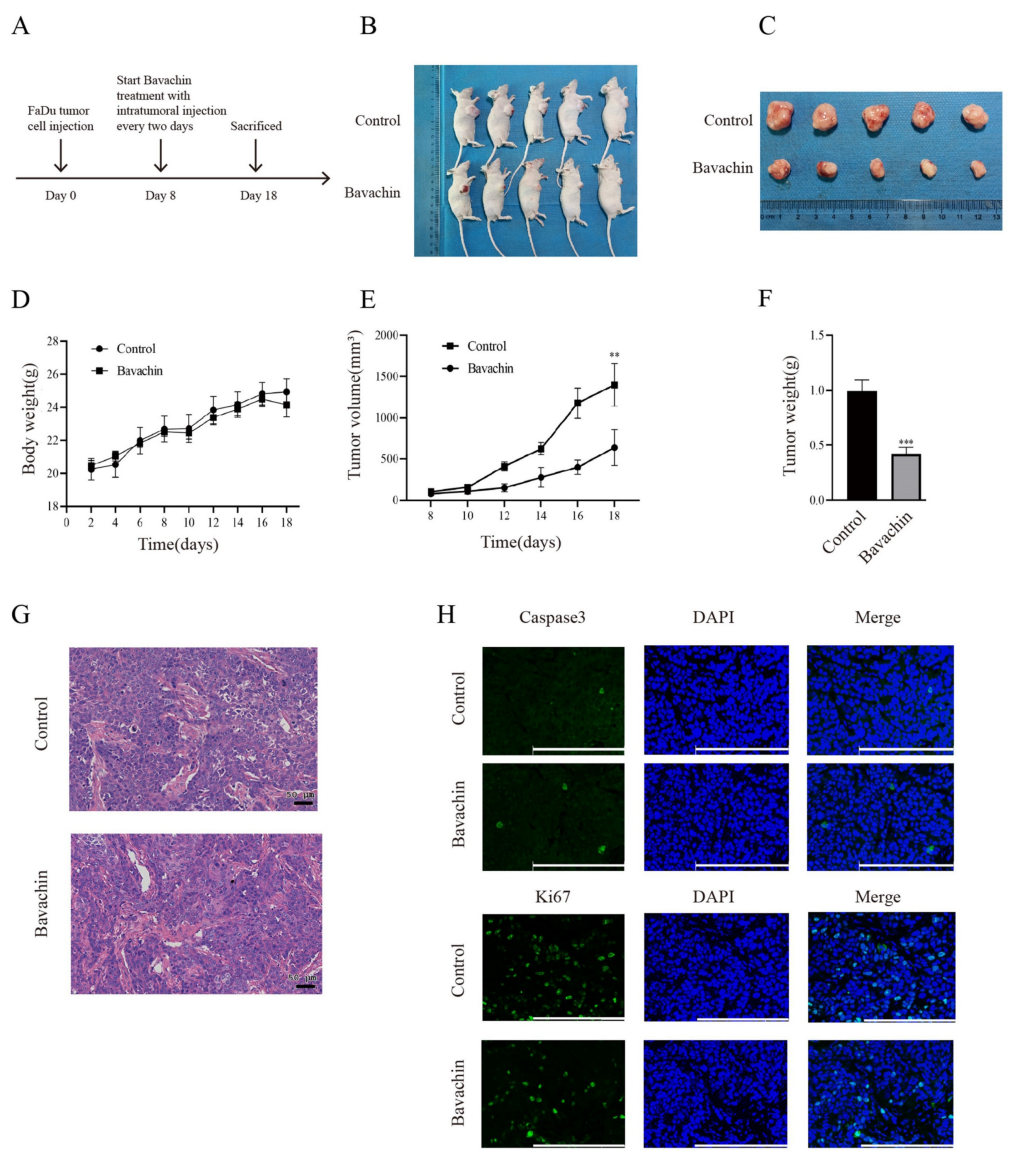
Bavachin inhibited tumor growth *in-vivo*. (A) Schematic diagram of the *in-vivo* experiment. (B-C) State of tumor growth in the human laryngopharyngeal cancer cells. The FaDu (4×10^6^) cells were injected into nude mice (n = 6). (D) Changes in mice body weight with time. (E) Changes in tumor volume with time. (F) Comparison of tumor weight between the experimental and control group. (G-H) Immunofluorescence staining of tumor tissues was performed for the detection of c-Caspase3 and Ki67. (Scale bar: 20 μm). *P <0.05, **P <0.01, ***P <0.001.

The FaDu cells (4×10^5^) were subcutaneously injected into the male BALB/c nude mice to further explore the suppressing effects of bavachin on the growth of laryngopharyngeal carcinoma cells *in vivo* (Figure 6. B-C). When tumor volume reached approximately 100 mm^3^, bavachin was injected at the tumor site after every two days (Figure 6. A). The results showed that the bavachin treatment markedly suppressed the tumor size and tumor weight as compared to the control group (Figure 6. E-F). Moreover, immunofluorescence analysis of the tumor tissues showed a significant decrease in the number of Ki67-positive cells in the experimental group as compared to the control group. Meanwhile, the proportion of cleaved-caspase3-positive cells was higher in the tumor tissues in the experimental group as compared to the control group (Figure 6. H). Collectively, these results indicated that bavachin could suppress the growth of human laryngopharyngeal cancer cells *in-vivo* by regulating the expression levels of Ki67 and cleaved-caspase3.

## Discussion

Laryngopharyngeal cancer is a common type of head and neck cancer in otorhinolaryngology. Although studies have focused on preventing laryngopharyngeal cancer, its incidence has been continuously increasing annually. Numerous therapeutic strategies have been designed for the treatment of laryngopharyngeal cancer, among which, surgical resection is the core treatment strategy. Nevertheless, the patients have a poor prognosis and impaired laryngopharyngeal function after surgery. Recently, increasing evidence suggested that traditional Chinese medicine and natural products might have significant function potential 22. Bavachin is a flavonoid extracted from the fruit of *Psoralea corylifolia* 14. Previous studies showed that bavachin exhibited positive antitumor effects through different mechanisms. For instance, bavachin could promote ROS generation and induce ferroptosis in the osteosarcoma cells by regulating the STAT3/P53/SLC7A11 signaling pathway 17. Additionally, bavachin could also suppress the growth of human placental choriocarcinoma cells and human hepatocellular carcinoma cells by causing mitochondrial dysfunction and endoplasmic reticulum stress 16, 23. Other studies reported that bavachin could induce the apoptosis of multiple myeloma cells by downregulating the expression levels of nuclear transcription factor-kappa B (NF-κB) and STAT3 and upregulating that of cleaved-caspase3 13. This study indicated that bavachin suppressed the growth of laryngopharyngeal cancer.

This study has demonstrated that bavachin could inhibit the proliferation of laryngopharyngeal cancer cells *in-vitro*. Moreover, bavachin could also significantly suppress the migration ability of laryngopharyngeal cells. Previous studies suggested that the loss of E-cadherin, an epithelial adhesion molecule, resulted in metastasis by breaking the intercellular contacts 24. Studies showed that the levels of N-cadherin, β-catenin, and MMP2 were markedly increased in the late tumor stages 18, 20. This study confirmed that the bavachin treatment could inhibit the expression level of N-cadherin, β-catenin, and MMP2 and increase that of E-cadherin in the laryngopharyngeal cancer cells.

Apoptosis is related to the mitochondrial and death receptor pathways 25. The mitochondrial apoptotic pathway is controlled by the Bcl-2 protein family, including pro-apoptotic proteins (Bax and Bad) and anti-apoptotic proteins (Bcl-2 and Bcl-xl) 26, 27. In the process of apoptosis, the fusion/fission proteins regulate the breakdown of mitochondria into fragments, causing cell death 28. Mitochondrial fusion is mainly mediated by three crucial proteins, including mitofusin-1 (MFN1), mitofusin-2 (MFN2), and optic atrophy 1 (OPA1) 29. This study showed that the bavachin treatment downregulated the protein expression levels of Bcl-2, MFN1, and MFN2 and upregulated that of Bax in the laryngopharyngeal cancer cells. These results indicated that bavachin could induce apoptosis by regulating the mitochondrial pathway.

The cell cycle arrest is correlated with apoptosis and regulation of cellular proliferation 30, 31. Numerous regulatory proteins are involved in the complex process of the cell cycle, among which, cyclin-dependent kinases (CDKs), including the cyclin proteins, are the main proteins 32. CDKs are the most vital regulatory proteins involved in the cell cycle, and the specific cyclins play important roles in the different phases of the cell cycle 33. In the current study, bavachin induced the arrest of Tu212 and FaDu cells at the G0/G1 phase, and significantly reduced the protein expression levels of CDK4/6, cyclin D1, and c-Myc. Therefore, the results suggested that bavachin could suppress the proliferation of Tu212 and FaDu cells by inhibiting the growth of cells entering the S stage of the cell cycle.

The MAPK family plays a crucial role in regulating the growth, differentiation, apoptosis, and stress response of the cells 34. The diverse stimuli can activate different MAPK pathways and regulate various biological processes; for instance, p38 and JNK are associated with oxidative stress response and cellular apoptosis 35. p38 and JNK regulate multiple transcription factors, leading to the upregulation of pro-apoptotic proteins and downregulation of anti-apoptotic proteins 36. A previous study showed that exogenous NO could induce the apoptosis of hepatocellular carcinoma cells by positive regulation of p38/JNK signaling pathways 37. Moreover, the STAT3 signaling pathway is also a vital intracellular signaling pathway, which mediates cellular growth and cycle 38. In general, the expression level of STAT3 is higher in tumor cells as compared to the normal cells and is beneficial for the proliferation of tumor cells and anti-apoptosis 39. This study showed that bavachin treatment downregulated the phosphorylation level of STAT3 and upregulated that of p38 and JNK in the Tu212 and FaDu cells. These results demonstrated that bavachin could suppress the proliferation of tumor cells and cause cell cycle arrest and apoptosis through the MAPK and STAT3 signaling pathways.

Previous studies reported that tumor cells had higher levels of ROS as compared to normal cells 40. Nevertheless, some chemotherapeutic drugs can cause oxidative stress injury and apoptosis of tumor cells by increasing the oxidative stress level in tumor cells 41. Recently, ferroptosis has been defined as the reaction of intracellular iron with H2O2, producing ROS and causing lipid peroxidation (LP) 42. In particular, the system xc- -GSH-GPX4-dependent antioxidant defense induces the accumulation of LP 43. A previous study reported that bavachin could induce ferroptosis in the osteosarcoma cells by inhibiting the expression level of STAT3 17 and inhibit human liver cancer cells by inducing apoptosis and concomitant accumulation of reactive oxygen species 44. Consistent, this study also showed that the bavachin-induced ferroptosis was correlated with the accumulation of ROS and consumption of GSH in the Tu212 and FaDu cells.

Altogether, bavachin could suppress the proliferation and migration of laryngopharyngeal cancer cells, resulting in the apoptosis and cell cycle arrest of the laryngopharyngeal cancer cells by regulating the MAPK/STAT3 signaling pathway. These results suggested a direct effect of bavachin on laryngopharyngeal cancer cells, which might be reversed by an antioxidant, thereby requiring further study. In the future, the invasion ability of bavachin on the laryngopharyngeal cancer cells should be further explored by constructing a lung metastasis model.

## Conclusions

Taken together, our findings suggested that bavachin could significantly inhibit the growth, migration, cell-cycle arrest, and induced apoptosis of laryngopharyngeal cells *in-vivo* and *in-vitro*. Furthermore, we also revealed that bavachin exhibited anti-tumor effects on laryngopharyngeal cells by regulating the MAPK and STAT3 signaling pathways. This study provided a basis for using bavachin as an anti-tumor agent for laryngopharyngeal cancer in the future.

## Figures and Tables

**Figure 7:**
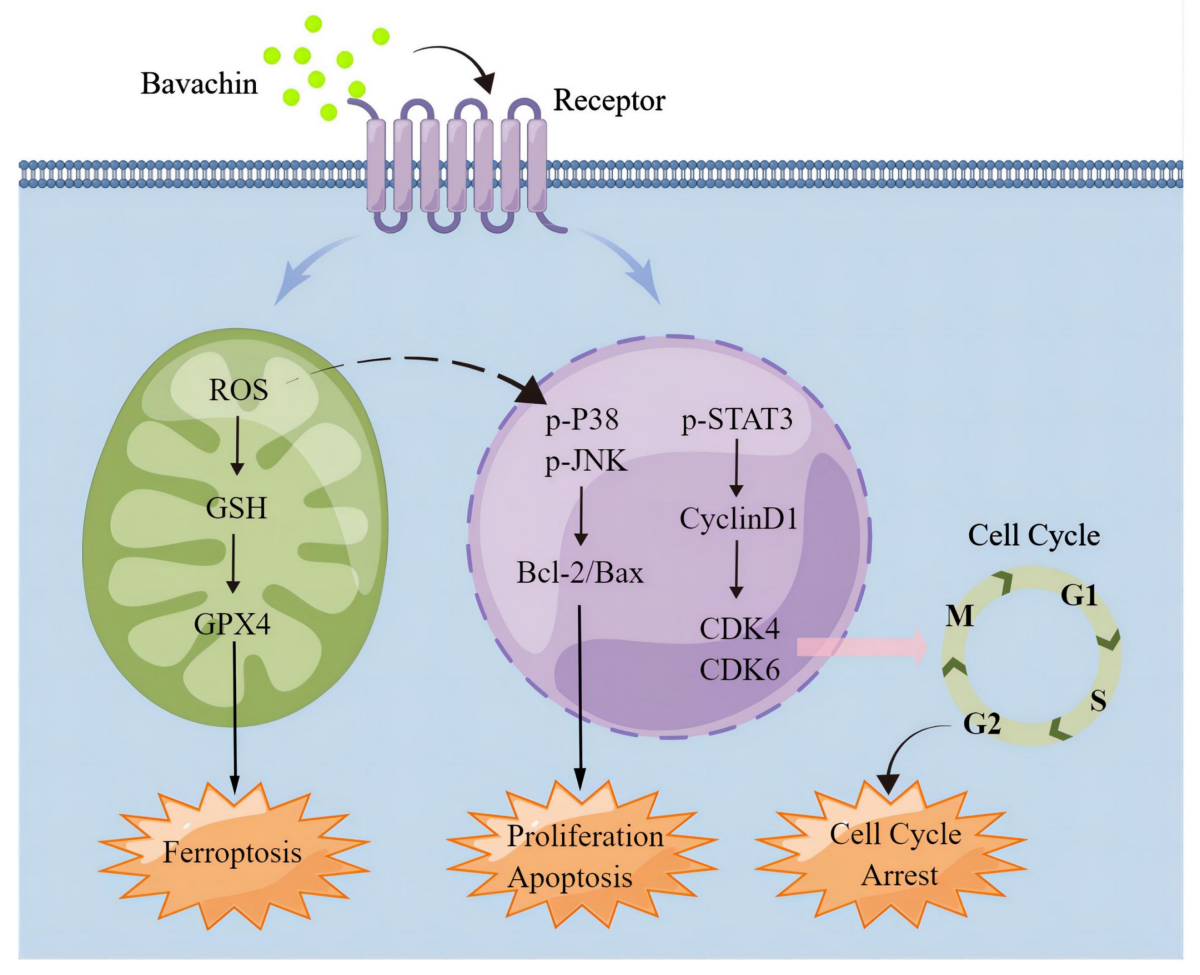
Schematic representation of the signaling pathways in laryngopharyngeal cancer cells affected by bavachin. This image was supported by Figdraw.

## Abbreviations

CDKs: cyclin-dependent kinases
DEPC: diethyl pyrocarbonate
EDTA: ethylenediamine tetraacetic acid disodium salt
GPX4: glutathione peroxidase 4
GSH: glutathione
HNSCC: head and neck squamous cell carcinoma
LP: lipid peroxidation
MAPK: mitogen-activated protein kinase
PBS: phosphate buffered saline
PI: propidium iodide
PVDF: polyvinylidene fluoride
ROS: reactive oxygen species
SPF: Specific pathogen Free
STAT3: signal transducer and activator of the transcription 3
